# Lessons from a Multilaboratorial Task Force for Diagnosis of a Fatal Toxoplasmosis Outbreak in Captive Primates in Brazil

**DOI:** 10.3390/microorganisms11122888

**Published:** 2023-11-29

**Authors:** Francine Bittencourt Schiffler, Asheley Henrique Barbosa Pereira, Silvia Bahadian Moreira, Igor Falco Arruda, Filipe Romero Rebello Moreira, Mirela D’arc, Ingra Morales Claro, Thalita de Abreu Pissinatti, Liliane Tavares de Faria Cavalcante, Thamiris dos Santos Miranda, Matheus Augusto Calvano Cosentino, Renata Carvalho de Oliveira, Jorlan Fernandes, Matheus Ribeiro da Silva Assis, Jonathan Gonçalves de Oliveira, Thayssa Alves Coelho da Silva, Rafael Mello Galliez, Debora Souza Faffe, Jaqueline Goes de Jesus, Marise Sobreira Bezerra da Silva, Matheus Filgueira Bezerra, Orlando da Costa Ferreira Junior, Amilcar Tanuri, Terezinha Marta Castiñeiras, Renato Santana Aguiar, Nuno Rodrigues Faria, Alzira Paiva de Almeida, Alcides Pissinatti, Ester Cerdeira Sabino, Maria Regina Reis Amendoeira, Elba Regina Sampaio de Lemos, Daniel Guimarães Ubiali, André F. A. Santos

**Affiliations:** 1 Laboratório de Diversidade e Doenças Virais (LDDV), Departamento de Genética, Instituto de Biologia, Universidade Federal do Rio de Janeiro, Rio de Janeiro 21941-617, RJ, Brazil; francine.schiffler@gmail.com (F.B.S.); mireladarc@gmail.com (M.D.); liliane.tavaresdefaria@gmail.com (L.T.d.F.C.); thamirismiranda02@gmail.com (T.d.S.M.); macosen@gmail.com (M.A.C.C.); 2 Setor de Anatomia Patológica (SAP), Departamento de Epidemiologia e Saúde Pública, Instituto de Veterinária, Universidade Federal Rural do Rio de Janeiro, Seropédica 23890-000, RJ, Brazil; asheleyhenrique@hotmail.com (A.H.B.P.); danielubiali@ufrrj.br (D.G.U.); 3 Centro de Primatologia do Rio de Janeiro (CPRJ), Instituto Estadual do Ambiente, Guapimirim 25940-000, RJ, Brazil; silviabm.inea@gmail.com (S.B.M.); alcidespissinatti@gmail.com (A.P.); 4 Laboratório de Toxoplasmose e outras Protozooses (LabTOXO), Instituto Oswaldo Cruz, Rio de Janeiro 21040-900, RJ, Brazil; igor_falco@yahoo.com.br (I.F.A.); amendoeira.fiocruz@gmail.com (M.R.R.A.); 5 MRC Centre for Global Infectious Disease Analysis, Abdul Latif Jameel Institute for Disease and Emergency Analytics (J-IDEA), Imperial College London, London SW7 2BX, UK; filipe.moreira@biologia.ufrj.br (F.R.R.M.); i.claro@imperial.ac.uk (I.M.C.); n.faria@imperial.ac.uk (N.R.F.); 6 Laboratório de Virologia Molecular (LVM), Departamento de Genética, Instituto de Biologia, Universidade Federal do Rio de Janeiro, Rio de Janeiro 21941-617, RJ, Brazil; orlandocfj@gmail.com (O.d.C.F.J.); atanuri1@gmail.com (A.T.); 7 Instituto de Medicina Tropical (IMT), Faculdade de Medicina, Universidade de São Paulo, São Paulo 05403-000, SP, Brazil; jaquelinegoesdejesus@gmail.com (J.G.d.J.); sabinoec@usp.br (E.C.S.); 8 Serviço de Criação de Primatas Não Humanos (SCPrim), Instituto de Ciência e Tecnologia em Biomodelos, Fundação Oswaldo Cruz, Rio de Janeiro 26382-462, RJ, Brazil; thalita.pissinatti@fiocruz.br; 9 Laboratório de Hantaviroses e Rickettsioses, Instituto Oswaldo Cruz, Rio de Janeiro 21040-900, RJ, Brazil; reoliveira@ioc.fiocruz.br (R.C.d.O.); jorlan@ioc.fiocruz.br (J.F.); matheus.assis@ioc.fiocruz.br (M.R.d.S.A.); goncalvesjohn03@gmail.com (J.G.d.O.); thayssa.silva@ioc.fiocruz.br (T.A.C.d.S.); elemos@ioc.fiocruz.br (E.R.S.d.L.); 10 Núcleo de Enfrentamento e Estudos de Doenças Infecciosas Emergentes e Reemergentes (NEEDIER), Universidade Federal do Rio de Janeiro, Rio de Janeiro 21941-599, RJ, Brazil; galliez77@gmail.com (R.M.G.); dfaffe@gmail.com (D.S.F.); tmartapc@gmail.com (T.M.C.); 11 Serviço de Referência Nacional em Peste, Instituto Aggeu Magalhães, Fundação Oswaldo Cruz, Recife 50740-465, PE, Brazil; marise.silva@fiocruz.br (M.S.B.d.S.); matheus.bezerra@fiocruz.br (M.F.B.); alzira.almeida@fiocruz.br (A.P.d.A.); 12 Laboratório de Biologia Integrativa, Universidade Federal de Minas Gerais, Belo Horizonte 31270-901, MG, Brazil; santanarnt@gmail.com; 13 Instituto D’OR de Pesquisa e Ensino (ID’or), Rio de Janeiro 22281-100, RJ, Brazil

**Keywords:** public policy, parasitic infection, toxoplasmosis, neotropical primate, one health, veterinary pathology

## Abstract

Toxoplasmosis is an important zoonotic disease caused by the parasite *Toxoplasma gondii* and is especially fatal for neotropical primates. In Brazil, the Ministry of Health is responsible for national epizootic surveillance, but some diseases are still neglected. Here, we present an integrated investigation of an outbreak that occurred during the first year of the COVID-19 pandemic among eleven neotropical primates housed at a primatology center in Brazil. After presenting non-specific clinical signs, all animals died within four days. A wide range of pathogens were evaluated, and we successfully identified *T. gondii* as the causative agent within four days after necropsies. The liver was the most affected organ, presenting hemorrhage and hepatocellular necrosis. Tachyzoites and bradyzoite cysts were observed in histological examinations and immunohistochemistry in different organs; in addition, parasitic DNA was detected through PCR in blood samples from all specimens evaluated. A high prevalence of *Escherichia coli* was also observed, indicating sepsis. This case highlights some of the obstacles faced by the current Brazilian surveillance system. A diagnosis was obtained through the integrated action of researchers since investigation for toxoplasmosis is currently absent in national guidelines. An interdisciplinary investigation could be a possible model for future epizootic investigations in animals.

## 1. Introduction

Between September and November 2020, during the first year of the Coronavirus Disease 2019 (COVID-19) pandemic and concomitant with the emergence of the P.2 strain in Brazil [1], eleven neotropical primates (NPs) from six different species died within approximately four days of the onset of clinical signs at the *Centro de Primatologia do Rio de Janeiro* (CPRJ) in Guapimirim, Rio de Janeiro, Brazil. Thus, in view of the reduced staff due to the mandatory quarantine and the official count of 141,406 deaths to that date, a health alert was issued to deal with the situation.

In Brazil, epizootic surveillance is overseen by the Brazilian Ministry of Health. A National List of Compulsory Notifiable Animal Diseases was created in 2006 and includes rabies, plague, influenza, and several arboviruses, but only gestational and congenital toxoplasmosis [2,3]. While reporting suspected or confirmed diseases present on the list is mandatory for all classes of animals, a majority of the reports are related to NPs, which are well-known sentinels for yellow fever virus (YFV) epizootics. This causes most surveillance guidelines to be aimed towards monitoring NPs and mosquitoes [4]. Consequently, when an NP dies, the standard procedure is to send samples to national surveillance reference laboratories specializing in YFV and RABV. However, if samples test negative for the compulsory notifiable diseases, the investigation is typically discontinued.

This is almost always what happens with cases of toxoplasmosis. Several cases have already been reported and reviewed around the world [5,6,7,8,9,10,11,12], and a vast majority have been acute and fatal, with nonspecific signs (mainly apathy, anorexia, abdominal distension, and fever), making diagnosis challenging [8,13,14,15,16,17]. Despite being on the list, as the risk to humans is restricted, current public policies do not cover its diagnosis in animals. In a scenario like Brazil, where the availability of public resources and personnel is narrowed, this becomes a limiting factor. Thus, in most cases, the animal dies without a diagnosis, or, when the result is available, there is no more time to apply the effective treatment.

Here, we present an integrated epidemiological and genomic investigation of this outbreak among captive NPs at the CPRJ/Brazil. A couple days after necropsies, we identified that toxoplasmosis was the cause of the deaths. It is important to highlight that the CPRJ serves as a scientific breeding facility for endangered NPs and is located in a rural area adjacent to the *Três Picos* State Park, encompassing approximately 160,000 acres of Atlantic Forest and housing 380 specimens from 29 species of NPs. Despite being in a national state of health emergency due to the pandemic, we mobilized a collaborative interdisciplinary network comprising research and public health laboratories to conduct a comprehensive investigation of the outbreak, including NPs and staff, testing for various pathogens.

## 2. Materials and Methods

### 2.1. Ethics Statement and Official Notification

All procedures conducted in this study adhered to the biosafety requirements established by the World Health Organization (WHO). Ethical approval for the study was obtained from the Ethics Committee on the Use of Animals in Scientific Experimentation at the Health Sciences Center from the *Universidade Federal do Rio de Janeiro* (CEUA-CCS/UFRJ), under protocol number 066/20. The collection and transportation of samples were further approved by the Biodiversity Authorization and Information System (SISBIO) of the *Instituto Chico Mendes de Conservação da Biodiversidade* (ICMBIO) (license number 75941-1). In addition, the present study received approval by the local ethics review committee at the *Hospital Universitário Clementino Fraga Filho* (*Certificado de Apresentação de Apreciação Ética*—CAAE: 30161620.0.0000.5257) and by the national ethical review board (CAAE: 30127020.0.0000.0068) for the collection of biological samples and testing of CPRJ workers. Regarding human sample collection, all participants included in the study were adults aged 18 years or older and provided consent through an informed consent form. CPRJ workers were tested for SARS-CoV-2 and *Yersinia* sp., as described below, and for Hepatitis A, B, and C (HAV, HBV, HCV); Cytomegalovirus (CMV); toxoplasmosis; and leptospirosis through the Brazilian Unified Health System. All information obtained during the outbreak investigation was made available to the Rio de Janeiro State Health Secretary through detailed and elaborate reports throughout the outbreak.

### 2.2. Animals

Our study focused on monitoring an outbreak that took place at the CPRJ between September and November 2020, resulting in the death of 11 captive NPs. During this period, all deceased primates underwent necropsy, including two individuals of *Brachyteles arachnoides*, three *Alouatta ululata*, two *A. guariba clamitans*, one *A. caraya*, two *Cacajao melanocephalus*, and one *Plecturocebus caligatus*. All procedures conducted during the necropsies were carried out in full compliance with and approved by the Brazilian Ministry of the Environment (SISBIO 30939-12).

All animals were fed the same basic diet, consisting of primate pelleted food and a mixture of vegetables and fruits. As a complement, insectivorous and omnivorous animals received additional sources of animal protein, and folivore animals received foliage purchased from local suppliers (mainly commercially grown vegetables, such as broccoli, kale, and chicory). All food was prepared in the same area and later distributed by the keepers in stainless-steel food containers, with each enclosure receiving an amount of food compatible with the number of animals present.

### 2.3. Sample Collection for Molecular Testing and Genetic Material Extraction

Since clinical signs appeared almost simultaneously in different NP species, the investigation was carried out in parallel in nine different laboratories, each employing different diagnostic methods until the pathogen was definitely identified. It should be noted that the order in which these methods were applied may vary.

A variety of samples were analyzed in this study, including blood and swab samples obtained from multiple specimens at different time points during the outbreak, as well as tissue fragments collected during necropsies. Blood samples from the femoral and saphenous veins of NPs (preferably) and by peripheral venipuncture from CPRJ staff were collected in EDTA tubes. For the serological analysis, serum was isolated via centrifugation at 400× *g* for 10 min. Swab samples were obtained by inserting sterile swabs into the selected cavity (rectal or oral for NPs, and nasopharyngeal for humans), rotated slightly, allowing for a 10-s period to absorb secretions, and then removed with slow circular movements. Swabs were stored with 1 mL of RNAlater^®^ (Invitrogen, Thermo Fisher Scientific, Waltham, MA, USA). Specimens were temporarily stored at room temperature and permanently stored at −80 °C.

Total nucleic acid extraction from swab and tissue samples was performed using the ReliaPrep™ Viral TNA Miniprep System (Promega, Madison, WI, USA). Swab samples were simply inverted in the tube and briefly centrifuged prior to extraction. Tissue extraction involved disrupting approximately 1 mm^3^ with 500 μL RNAlater^®^ solution, using Lysing Matrix E (MP Biomedicals do Brasil, São Caetano do Sul, SP, Brazil) on a Super FastPrep-2 (MP Biomedicals, Valiant, CN, USA) through 30-second cycles, alternating with an ice bath until complete dissolution. The mixture was then centrifuged at 6500× *g* at 4 °C, and 200 μL of the supernatant was used, following the manufacturer’s protocol. Nucleic acid extraction from blood samples was performed using the MasterPure™ Complete DNA and RNA Purification Kit (Lucigen, LGC Ltd., Teddington, UK). For the metagenomic analysis, samples were centrifuged for 5 min at 10,000× *g* before extraction.

### 2.4. Necropsy and Histopathology

As the animals died, they were subjected to necropsy by the veterinary pathologists at the *Setor de Anatomia Patológica* from *Universidade Federal Rural do Rio de Janeiro* (SAP/UFRuralRJ), together with members of the CPRJ and the *Serviço de Criação de Primatas Não-humanos* from *Instituto de Ciência e Tecnologia em Biomodelos* (SCPrim ICTB). A total of 11 NPs underwent the procedure: 5 were necropsied immediately after death, and 5 were necropsied the day after death, with the carcasses being kept under refrigeration until the procedure; the last animal was then necropsied shortly after euthanasia. All procedures were performed using personal protective equipment (PPE) compatible with Biosafety Level 3 (BSL-3), including specific lab coats, gloves, and eye protection. During necropsies, organs (skin, brain, lymph nodes, lung, heart, trachea, esophagus, thyroid, adrenal, spleen, kidney, liver, stomach, and intestines) were collected in 10% buffered formalin and fixed 24–48 h for routine histological processing. Fragments of approximately 1 cm^3^ were also excised and stored with 1 mL of RNAlater^®^ until nucleic acid extraction. Standardized brain 5-section trimming was performed, corresponding to the following structures: telencephalon, hippocampus, amygdaloid nuclei, diencephalon, mesencephalon, ventricular system, *cornu ammonis*, cerebellum, pons, and myelencephalon. Sections were stained with hematoxylin and eosin (HE) for optical microscopy.

### 2.5. Detection of Viral Agents

#### 2.5.1. SARS-CoV-2

All procedures were performed by the Laboratório de Diversidade e Doenças Virais (LDDV), the Laboratório de Virologia Molecular (LVM), and the Núcleo de Enfrentamento e Estudos de Doenças Infecciosas Emergentes e Reemergentes (NEEDIER) from UFRJ. Genetic material extracted from nasopharyngeal swabs from CPRJ workers and oral swabs and tissues (lung, trachea, and intestine) from NPs were used for molecular detection in a one-step reverse transcription–quantitative polymerase chain reaction (RT-qPCR) system, using GoTaq^®^ Probe qPCR Master Mix (Promega, Madison, WI, USA) and the CDC 2019-Novel Coronavirus (2019-nCoV) Real-Time RT-PCR Diagnostic Panel (Integrated DNA Technologies, Coralville, IA, USA), according to the manufacturer’s instructions. This protocol targets the SARS-CoV-2 N1 and N2 genes and, as an internal control, the human ribonuclease P (RNaseP) gene. All reactions were performed in a 7500 Real-Time PCR System (Applied Biosystems, Waltham, MA, USA). Samples were considered positive when both targets (N1 and N2) amplified with cycle threshold (Ct) ≤  37.

For serological diagnosis, an Enzyme-Linked Immunosorbent Assay (ELISA) protocol was performed for both staff members from CPRJ and NP. The 96-well ELISA plates (Corning Inc., Somerville, MA, USA) were coated overnight at 4 °C with 200 ng per well of the SARS-CoV-2 protein S produced by the *Laboratório de Engenharia de Cultivos Celulares* (LECC) from the UFRJ by Prof. Leda Castilho [18]. After a cycle of five washes with phosphate-buffered saline (PBS) 0.05% Tween-20, the plates were blocked with 100 µL of 5% Bovine Serum Albumin (BSA) and incubated for 2 h at room temperature. Each serum sample was tested at a dilution of 1:50 in PBS 0.05% Tween-20, with 2% BSA and Bromocresol Purple added to the wells, and incubated for 1 h at 37 °C. After a new cycle of five washes, 50 μL of horseradish peroxidase (HRP)-conjugated goat anti-Monkey IgG antibody (1:10,000, Thermo Fisher Scientific, USA) was added to each well, and the plate was then incubated at 37 °C for 1 h, followed by a new cycle of washes. Lastly, 50 μL of TMB (3,3′,5,5′-tetramethylbenzidine) substrate (Thermo Fisher Scientific, Waltham, MA, USA) was added into each well. After 10 min of incubation, the reaction was stopped by adding 50 μL of 1 M H_2_SO_4_ solution. Reactions were analyzed at a 450 nm wavelength, and the results were expressed in optical density (OD). All samples were tested in duplicate and, therefore, were considered reactive when the mean of the ODs of the replicates exceeded the cutoff, obtained by calculating the mean of the ODs of the negative controls plus three times the standard deviation. To normalize the OD values between reaction plates, the percentage of the difference between the OD of the well with sample and the average OD of the white wells (only with the protein) was calculated.

To evaluate the presence of coronaviruses other than SARS-CoV-2, samples of the lungs and trachea of necropsied NPs, as well as blood samples from sick NPs were used for PCR amplification with a set of PanCoronavirus (PanCov) primer sequences (α-, β-, γ-, and δ-coronaviruses) [19]. After RNA extraction, the synthesis of cDNA was performed with the High-Capacity cDNA Reverse Transcription Kit (Thermo Fisher Scientific, Waltham, MA, USA), followed by PCR reaction with Platinum™ Taq DNA Polymerase (Invitrogen, Thermo Fisher Scientific, Waltham, MA, USA). This reaction consisted in 2.5 μL of 10× PCR Buffer (1×), 1 μL of 50 mM MgCl_2_ (2 mM), 0.2 μL of 25 mM dNTP (0.2 mM), 1 μL of each primer (150 pmol), 0.25 μL Platinum™ Taq DNA Polymerase (1.25 U), 5 μL cDNA template, and 15 μL Nuclease-Free Water. The reaction was conducted with an initial activation at 94 °C for 2 min, followed by 35 cycles of amplification (30 s at 94 °C, 5 min at 52 °C, and 1 min at 72 °C) and a final extension step at 72 °C for 1 min. The results were visualized with a 1% agarose gel electrophoresis.

The Panbio COVID-19 Ag Rapid Test Device (Abbott Rapid Diagnostic Jena GmbH, Jena, TH, DE) was also used to detect the viral nucleocapsid protein in nasopharyngeal samples from CPRJ workers. Detection was performed immediately after sampling, following the manufacturer’s instructions (reading up to 15 min). For NP testing, only oral samples were used, since the small size of the animals prevented the collection of nasal and nasopharyngeal samples. Lastly, to completely rule out a SARS-CoV-2 infection in NPs, veterinarians from the SAP/UFRuralRJ further tested lung the samples of necropsied NPs via immunohistochemistry (IHC), using an Anti-Sars-CoV Nucleocapsid Protein (Novus Biologicals^®^, Centennial, CO, USA, catalog no. NB100-56576) [20].

#### 2.5.2. Arenavirus and Hantavirus

Serum samples of NPs were screened for IgG antibodies against recombinant nucleoprotein protein (rN) of *Orthohantavirus andesense* (*Mammantavirinae*: *Hantaviridae*) and *Mammarenavirus choriomeningitidis* (LCMV; *Arenaviridae*) whole proteins by an ELISA protocol, as previously described [21,22] by the *Laboratório de Hantaviroses e Rickettsioses* (LHR) from the *Instituto Oswaldo Cruz* (IOC) of the *Fundação Oswaldo Cruz* (Fiocruz).

To evaluate the presence of arenavirus and hantavirus RNA, RNA from blood and tissue samples from NPs were used for RT-PCR amplification, using SuperScript™ IV One-Step RT-PCR System kit (Invitrogen, Thermo Fisher Scientific, Waltham, MA, USA) with a set of primer sequences targeting hantavirus nucleoprotein and polymerase genes [23,24], arenavirus glycoprotein [25], and lymphocytic choriomeningitis virus (LCMV) polymerase gene [26]. The results were visualized in a 1.5% agarose gel electrophoresis.

#### 2.5.3. Arboviruses

Blood samples from NPs were also screened for arboviruses in the State Reference Laboratory. After RNA extraction, samples were subjected to a RT-PCR, as previously described [27], which targets the highly conserved 5′-noncoding region (5′-NC) of the yellow-fever virus (YFV) genome. Samples were considered positive when presenting a threshold cycle value ≤ 37. Furthermore, the presence of other main circulating arboviruses was evaluated via RT-qPCR, using GoTaq^®^ 1-Step RT-qPCR System (Promega, Madison, WI, USA). Specific diagnosis primers were used for Zika [28], dengue [29], chikungunya [30], Mayaro [31], West Nile [32], and Oropouche [33] viruses. Reactions were assembled following the manufacturer’s instructions and were performed in a 7500 Real-Time PCR System (Applied Biosystems, Waltham, MA, USA).

### 2.6. Metagenomic Sequencing

To investigate the presence of pathogens in an unbiased fashion, we performed rapid metagenomic sequencing, using portable Oxford Nanopore sequencing Technology. A total of 20 NP samples from four different tissues were selected for metagenomic sequencing primarily based on histopathological alterations (liver and intestine, n = 9), followed by organs from the respiratory tract (due to the suspicion of respiratory virus infection) (lung, n = 4). For those for which no tissue sample was collected, we sequenced only blood samples (n = 7). Each sample was analyzed separately in a different sequencing library.

After extraction, isolated RNA was transported to the *Instituto de Medicina Tropical* at *Universidade de São Paulo* (IMT/USP), where sequencing libraries were prepared and sequenced following the SMART-9N protocol [34]. Raw FAST5 files were basecalled using Guppy software version 2.2.7 GPU basecaller (Oxford Nanopore Technologies, Oxford, Oxon, UK) and then demultiplexed using the same software. We performed taxonomic classification, using Kraken v. 2.0.7_beta [35], with the miniKraken_v2 database. Interactive visualization plots were generated with Krona v. 2.8.1 [36]. Afterwards, barcoded FASTQ files were mapped to reference genomes, using MiniMap2-2.17 (r941) [37]. Tablet v. 1.19.05.28 [38] was used to visualize mapping files, count mapped reads, and calculate the percentage of genome coverage and sequencing depth.

### 2.7. Bacterial Agents

#### 2.7.1. *Yersinia pestis*

Because the location where the outbreak occurred overlaps with a known bubonic plague foci [39], sera from the subjects were evaluated for the presence of the *Yersinia pestis*-specific F1 capsular antigen pestis antibodies. For confirmatory purposes, two distinct multi-specie approaches were used: hemagglutination and ELISA-Protein A. The serology tests were performed by the Plague National Reference Service at the *Instituto Aggeu Magalhães* (IAM)—*Fiocruz*, and the detailed protocol is described elsewhere [40].

To evaluate the presence of *Y. pestis*, bone marrow and sera samples from all NPs were submitted to bacterial culture. Furthermore, the spleen, lungs, kidney, and whole blood of one individual (*A. caraya*, ID 2576) were also submitted to bacterial culture. After sample collection, the cleared phalange bones were sent in a sterile tube to the BSL-3 laboratory in the IAM. The bone was sprayed with ethanol 70% at the external parts, and the head of the bone was removed. The bone marrow was collected with a syringe, diluted 1:1 parts in sterile saline solution and with a bacteriological loop, and was transferred to the following media: blood agar base (BAB), agar MacConkey, and agar Salmonella–Shigella. The plates were incubated at least for 48 h at 28 °C and the colonies were tested using the *Y. pestis*-specific bacteriophage-lysis test [41], multiplex PCR (as described below), and API 20E gallery (Biomérieux, Marcy-l’Étoile, France).

Molecular detection of *Y. pestis* was performed using an in-house multiplex PCR, using four primer sets. The primers targeted regions of the *caf1*, *pla*, and *lcrV* genes located on the pFra, pPst, and pYV plasmids, respectively, and the *irp2* chromosomal gene [42]. The PCR products were visualized in 1% agarose gel stained with SYBR safe (Thermo Fisher Scientific, Waltham, MA, USA).

#### 2.7.2. *Escherichia coli*

The lung sections of 11 NPs were tested using the IHC technique with an anti-*E. coli* (Rabbit Antibody to *E. coli*, catalog number 1001, Virostat^®^, Westbrook, ME, USA) for sepsis investigation, following standardized protocols [43].

### 2.8. Protozoan Agents

#### Toxoplasma gondii

The lungs and liver sections of 11 NPs were submitted to the IHC technique with an anti-*T. gondii* (Dako, Carpinteria^®^, CA, USA), using standardized protocols [44]. Serum samples of seven NPs (three *A. ululata*, two *A. guariba*, one *A. caraya*, and one *B. arachnoides*) were subjected to an indirect fluorescent antibody test (IFAT) in the *Laboratório de Toxoplasmose e outras Protozooses* (LabTOXO), IOC/Fiocruz. Following Camargo (1964), IgG anti-*T. gondii* antibody detection was conducted using an anti-monkey IgG FITC conjugate produced in rabbit (Sigma-Aldrich^®^, St. Louis, MI, USA). *T. gondii* RH-strain tachyzoites, which were maintained in Swiss Webster mice, were used as antigens. The follow-up of IgG anti-*T. gondii* titers was performed only in the surviving black-and-gold howler monkey (*A. caraya*, ID 2576) in the following five weeks post clinical signs’ onset. A serum sample of a Colombian red howler monkey (*A. seniculus*) with detectable antibodies by MAT (1:4096) was used as positive control [45]. In view of the positive result for NPs, an employee from CPRJ who was pregnant at the time of the outbreak was also tested for the pathogen.

Whole blood samples from the same seven animals tested in serology were used to detect *T. gondii* DNA. DNA extraction was performed using the Qiamp DNA Blood Mini Kit (Qiagen^®^ Inc., Hilden, Germany), following the manufacturer’s instructions. *T. gondii* DNA detection was performed using conventional PCR to amplify a 529 base pair (bp) repeat element (REP529) previously described [46]. DNA amplification was observed by electrophoresis in agarose 1% gel stained with GelRed^®^ (Biotium, Fremont, CA, USA). The DNA from *T. gondii* RH strain tachyzoites was used as the positive control. The produced amplicons were purified by cycling with ExoSAP-IT enzyme (Applied Biosystems, Waltham, MA, USA). All the samples were sequenced using the same primers as those used in the PCR reactions, in a 3730xl DNA analyzer (Applied Biosystems, Waltham, MA, USA). Sequences were analyzed through Bioedit v.7.1.9 [47] and compared with the NCBI database, using the BLASTn tool v5 (https://blast.ncbi.nlm.nih.gov/Blast.cgi; accessed on 20 March 2022).

## 3. Results

### 3.1. Clinical Description of the CPRJ Outbreak in Neotropical Primates

**Figure 1:**
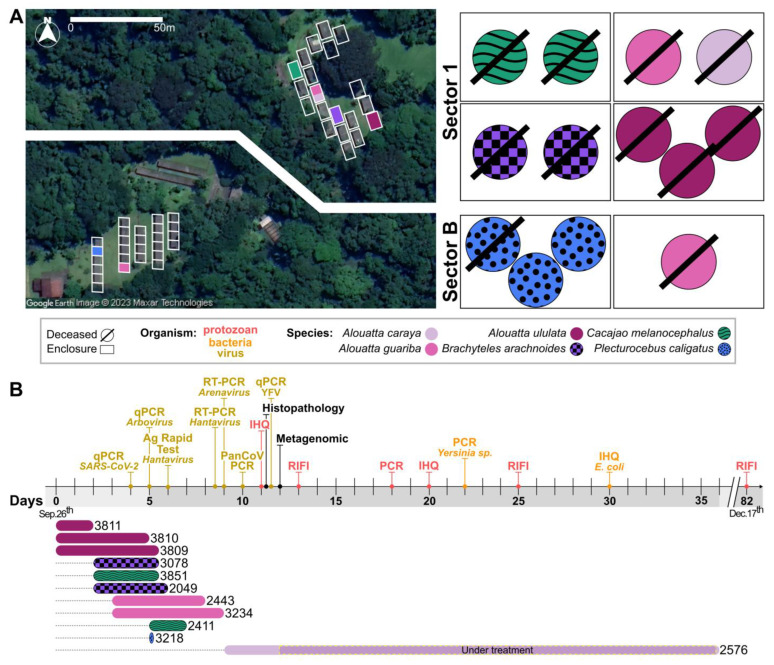
Geographical distribution of the cases and general course of the outbreak. (**A**) Spatial location of the enclosures (**left**) and their graphical representation (**right**). The rectangles represent the enclosures, which are colored according to the neotropical primate (NP) species affected by the outbreak: *Alouatta* genus in different shades of pink, *Brachyteles* in orange, *Cacajao* in green, and *Plecturocebus* in blue. Unfilled rectangles had no animals affected by the outbreak. (**B**) The top half of the timeline chronologically shows all major tests performed during the outbreak, separated by color according to the pathological agent: protozoan (*Toxoplasma gondii*) in red, bacterial agents in orange, and viral agents in dark yellow. The exceptions are the metagenomic sequencing and histopathology, which are highlighted in black. Actual timeline is represented as a horizontal bar in consecutive days from the onset of symptoms in the first primate (day 0) until the death of the last animal (day 82). Double bars represent a time cut. The bottom half of the display is dominated by the graphic representations of the symptomatic periods of each animal. The bars represent the beginning of the clinical signs until the death of the respective primate. Numbers on the right side of each bar represent the animal identification, and colors and patterns inside the bars represent the different NP genera. Only one animal (3218) died without showing any clinical sign.

Eleven primates from six different species housed in six enclosures in two sectors of the CPRJ were affected by the outbreak from September to November 2020 (Figure 1A). One species is listed as critically endangered (*B. arachnoides*), and one is listed as endangered (*A. ululata*) on the Red List of Threatened Species by the International Union for Conservation of Nature and Natural Resources (IUCN). One primate (female *P. caligatus*) did not show clinical signs and was found dead a few hours after normal feeding. Ten primates presented at least one clinical sign, and two of them (male and female *C. melanocephalus*) died before a complete clinical examination could be performed. Inappetence and anorexia were the first clinical signs to be observed in symptomatic animals and were observed in all of them. Prostration was also observed in all symptomatic individuals, ranging from mild (lethargy, but still moving around the enclosure—1/10 animals; 10%), moderate (animal visibly depressed, spending most of the time motionless, but still perched—7/10 animals; 70%), and accentuated (animal totally lethargic, unable to perch, remaining on the enclosure floor—2/10 animals; 20%). Two primates (20%) showed drowsiness and an inability to keep their eyes open.

Abdominal pain and distension were observed in 20% and 40% of symptomatic primates, respectively. Respiratory alterations, such as dyspnea and wheezing, on pulmonary auscultation were present in 20% of the symptomatic animals, while nasal secretion was observed in only one primate (10% of the symptomatic ones). Body temperature was measured in 8 of the 11 affected animals and ranged from 36.2 to 41.4 °C (97.16 F to 106.52 F). For ten of the eleven animals (91%), the time between the onset of symptoms and death ranged from zero to seven days with an average time of 3 days. One animal survived longer (36 days) and was later euthanized due to poor prognosis (for details, see [48]). The general course of the outbreak and the main testing are both summarized in Figure 1B.

### 3.2. Pathological Description of the CPRJ Outbreak in Neotropical Primates

**Figure 2:**
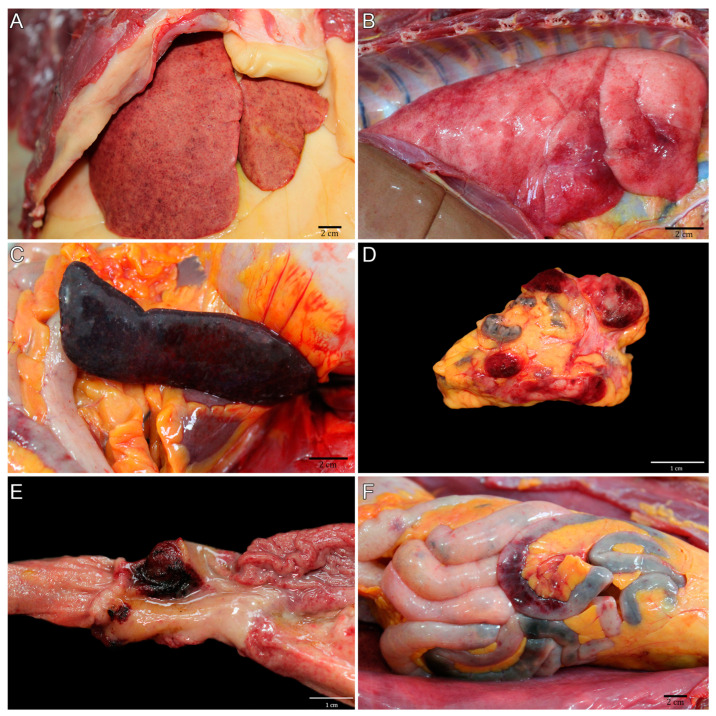
Gross findings of toxoplasmosis in neotropical primates (NPs). (**A**) NP 2, liver diffusely enlarged, with random hepatocellular necrosis and hemorrhage areas. (**B**) NP 5, multifocal hemorrhagic areas on pleural surface. (**C**) NP 11, diffuse splenomegaly with white multifocal dots at the capsular surface. (**D**) NP 11, mesenteric lymph node enlarged with multifocal white and red areas at the cut surface. (**E**) NP 11, evident focal ulcer in the pylorus. (**F**) NP 11, petechial-to-ecchymotic multifocal areas at the jejunum serosal surface.

The main pathological findings are described in Table 1 and were carried out by a multi-institutional team formed by members of CPRJ, SAP/UFRuralRJ, and SCPrim/ICTB. The liver of the 11 NPs was the hallmark gross organ. It was markedly enlarged, with white-to-yellow random dots (hepatocellular necrosis) mottled with random red dots (hemorrhage) at the capsular (Figure 2A) and cut surface. In the lungs, the pleural surface was irregularly covered by multifocal red areas (hemorrhage) (Figure 2B). White multifocal dots were seen at the spleen’s capsular and parenchyma cut surface (Figure 2C). Mediastinal and mesenteric lymph nodes were enlarged, and the cut surface contained multifocal white and red areas (Figure 2D). The stomach showed marked focal ulcerative gastritis (Figure 2E). The jejunum showed petechial-to-ecchymotic multifocal areas at the serosal surface (Figure 2F). With the presented scenario, an epizootic notification was made to the State Department of Health of Rio de Janeiro, as officially required.

### 3.3. Negative SARS-CoV-2 and Arboviral Diagnoses

As the first affected NPs had clinical respiratory signs, and considering the COVID-19 pandemic scenario in Brazil, we first conducted SARS-CoV-2 testing in three laboratories from UFRJ (LDDV, LVM, and NEEDIER). We obtained negative molecular diagnostic results for primates and CPRJ workers. The serological results were also negative for all NPs, but they were positive for two animal keepers, consistent with previously undiagnosed SARS-CoV-2 infection. Additional molecular testing for universal diagnosis of coronavirus was also performed on swab and tissue samples from primates, again with negative results (Figure 1B).

In view of the pathological findings, especially the intestinal hemorrhages, a multiplex qPCR test for seven arboviruses (YFV, dengue, Zika, chikungunya, Mayaro, Oropouche, and West Nile) was performed, all with negative results, which were confirmed by the Regional Reference Laboratory for Yellow Fever of IOC. Following investigation, NP blood samples were forwarded to the reference LHR/IOC—Fiocruz for hantavirus and arenavirus investigation, including lymphocytic choriomeningitis virus, and the samples were all negative.

Concomitantly, recommendations regarding biosafety issues were made to ensure the safety of the CPRJ workers and NPs, as well as the scheduling of clinical care and collection of blood samples for additional analysis of all professionals with a clinical condition or with a history of contact with animals. A 10-week pregnant worker had no clinical manifestation, while four other workers had mild influenza-like clinical symptoms. The serological tests for toxoplasmosis and hepatitis A, B, and C were all non-reactive.

### 3.4. Histological Findings for Neotropical Primates

**Figure 3:**
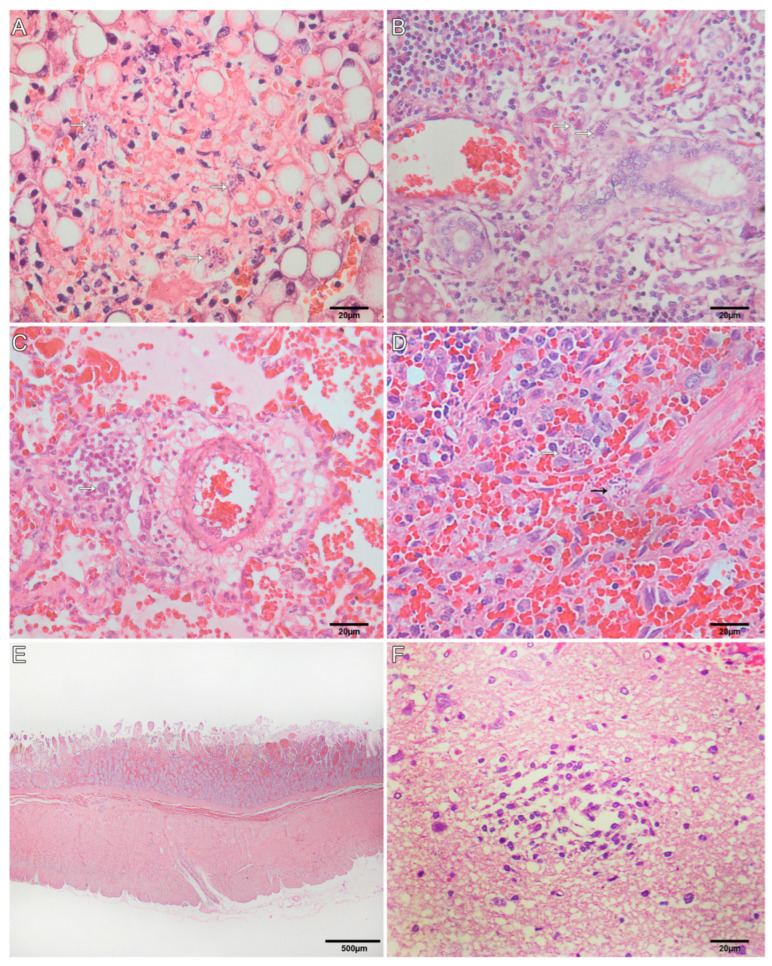
Histological findings of toxoplasmosis in neotropical primates (NPs). (**A**) NP 5, marked hepatocellular necrosis with intralesional tachyzoites (HE, Obj. 40×). (**B**) NP 5, lymphocytic periportal hepatitis with intralesional cysts of bradyzoites (white arrow) (HE, Obj. 40×). (**C**) NP 11, alveolar septa expanded by lymphocytes and intralesional cysts of bradyzoites (white arrow). There are multifocal areas of edema and hemorrhage in the adjacent airways (HE, Obj. 40×). (**D**) NP 2, necrotizing splenitis with intralesional cysts of bradyzoites (white arrow) and tachyzoites (black arrow) (HE, Obj. 40×). (**E**) NP 11, duodenal mucosa and submucosa expanded by marked hemorrhage (HE, Obj. 2.5×). (**F**) NP 9, focal malacia area at the telencephalic cortex gray matter (HE, Obj. 40×).

Histological examinations were performed by SAP/UFRuralRJ, and the findings are described in Table 2. Extracellular structures, rounded to fusiform, basophilic, with 2-to-3 µm (tachyzoites); and basophilic, thin-walled oval structures, with an average of 20 × 15 µm, filled with elongated basophilic bradyzoites ranging in size from 1 to 2 µm (bradyzoite cysts) were visualized. In the liver, there was multifocal random hepatocellular necrosis and lymphocytic periportal hepatitis with intralesional tachyzoites (Figure 3A) and cysts of bradyzoites (Figure 3B), marked multifocal lipidosis, and multifocal hemosiderosis. Necrotizing fibrinoid vasculitis was observed in the liver of two primates. In the lung, there was multifocal thickening of alveolar septa by lymphocyte infiltration, intra-alveolar foamy macrophages, alveolar edema and hemorrhage, necrosis of type I pneumocyte, and fibrin deposition in the alveolar space, occasionally forming a hyaline membrane. Intralesional cysts of bradyzoites and tachyzoites were seen (Figure 3C). Necrotizing splenitis with neutrophilic and histiocytic infiltration was seen in the red and white pulp, fibrin deposition, and intralesional cysts of bradyzoites and tachyzoites (Figure 3D). The lymph nodes showed medullary-to-cortical necrosis, neutrophil, and histiocyte infiltration, mainly at the subcapsular sinus, with intralesional cysts of bradyzoites and tachyzoites. Necrohemorrhagic duodenitis with intralesional cysts of bradyzoites and tachyzoites (Figure 3E) was seen in three primates. Three primates presented brain lesions characterized by multifocal areas (telencephalon gray and white matter, corpus callosum, cerebellum molecular layer, and myelencephalon) of malacia with intralesional tachyzoites (Figure 3F). In one case, the neuronal lesions were characterized by multifocal gliosis at telencephalon gray and white matter. Necrohemorrhagic typhlitis with intralesional cysts of bradyzoites and tachyzoites was seen in one primate. Another primate showed lymphocytic interstitial nephritis.

### 3.5. Pathogen Metagenomics Reveals Sepsis Causing Bacterial Infection

In light of the emergency for a diagnosis and the need for a more detailed investigation, untargeted metagenomic sequencing was performed in a partnership between LDDV/UFRJ and IMT/USP. All samples analyzed presented a high proportion of unclassified reads, corresponding to approximately 67% (min, 58%; and max, 85%) of the total reads generated (Supplementary Figure S1). The remaining reads, in most libraries (n = 13/20), were identified as eukaryotes (Homo sapiens), followed by bacteria and viruses. Seven libraries (n = 7/20) showed a higher proportion of bacterial reads, representing up to 26% of all reads generated in each library. For these samples, an inversion in taxonomic representativeness was observed, with bacteria being the most represented taxonomic level, followed by eukaryotes and viruses.

Regarding the identified bacteria, diversity varied considerably between samples, without an overall dominant order or family. Nonetheless, the presence of some bacteria caught our attention, such as *E. coli* and the *Yersinia* genus. Although *E. coli* is naturally found in the intestine, it is not usually found in other organs. It is important to point out that more than half of these libraries (57%; 4/7) were generated from plasma samples, a tissue that, in theory, should be sterile. This observation, together with the necropsy findings, led us to conclude that these animals suffered from a bacterial infection that caused sepsis.

Following the histopathology observation of different *T. gondii* forms, we performed a reference assembly with the metagenomic data (reference accession number: NC_031467.1), which confirmed the presence of this pathogen. We found over 660,000 reads across all samples (median number of mapped reads: 33,000) and up to 109,870 reads in just one liver sample (from 2.5 to 17 times more than other tissue samples) (Supplementary Table S1).

### 3.6. Immunohistochemistry

**Figure 4:**
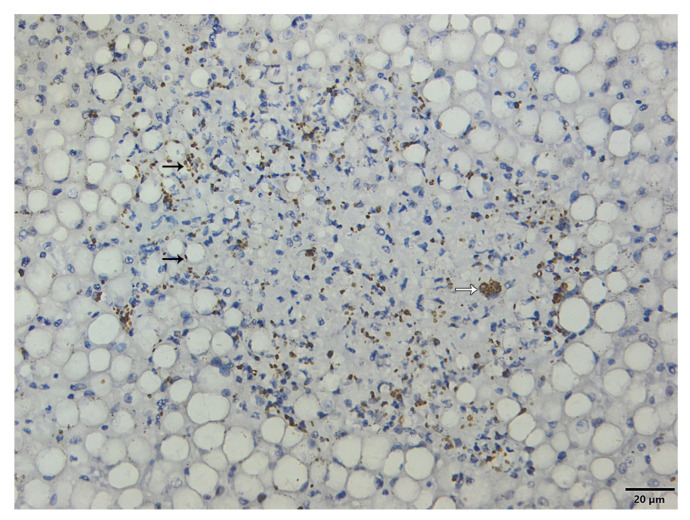
Immunohistochemistry using an anti-Toxoplasma gondii antibody. Bradyzoites cysts (white arrow) and tachyzoites (black arrow) are immunolabeled within hepatocellular necrosis focus (IHC, Obj. 40×).

The immunohistochemistry was performed by SAP/UFRuralRJ, and the findings are summarized in Table 3. Intralesional cysts of bradyzoites and tachyzoites were visualized in more than one organ of almost all primates that died naturally (10/11; Figure 4). In only one case of *A. caraya* (#8, ID 2576) with subacute evolution, *T. gondii* structures were not seen. The clinical–pathological findings were already reported [49]. Infection by SARS-CoV-2 by IHQ was ruled out in all 11 tested NPs. Out of eleven primates, seven were tested for *E. coli* infection and confirmed for coinfection of *T. gondii* and *E. coli* pneumonia, as well as sepsis, which was confirmed by vessels intraluminal *E. coli* immunolabelling and contributed to the worsening of their health conditions.

### 3.7. Molecular and Serological Diagnosis of Toxoplasmosis

In view of the positive result for toxoplasmosis, blood samples from all animals were also tested molecularly in the LabTOXO/IOC—Fiocruz. Out of the seven serum samples of NPs initially submitted to IFAT, only the sample from a black-and-gold howler monkey (*A. caraya*, ID 2576) showed IgG anti-*T. gondii* (1/7, 14.3%), with titers of 1:16. The serological follow-up of this animal showed a progressive increase in antibody titers in the five consecutive weeks, reaching titers of 1:64 and 1:256 in the second and fourth weeks of illness, respectively. Additionally, the 529 bp repeat element of *T. gondii* DNA was detected in all whole blood samples submitted to PCR. Of these, six showed high identities with *T. gondii*, ranging between 98% and 100%, when compared with nucleotide sequences deposited in GenBank (LC547467.1). Therefore, with the clinical–pathological presented picture and the multiple auxiliary examinations for *T. gondii* detection, we concluded that toxoplasmosis was the most likely etiological agent causing this outbreak.

### 3.8. Negative Yersinia Bacteriological, Molecular, and Serological Diagnosis

Regarding the *Yersinia* genus, we found metagenomic reads from different species in all samples submitted to metagenomics. A reference-guided assembly was conducted with *Y. pestis* (AE017042.1), *Y. enterocolitica* (NC_008800.1), and *Y. pseudotuberculosis* (NZ_LR134373.1) complete genomes, some of the main *Yersinia* of zoonotic importance. We found that *Y. pestis* recovered the least amount of reads in all samples (approximately 228,000 reads total; max, 40.532; and min, 1.229). On the other hand, the assembly using *Y. enterocolitica* genome as the reference was the most effective, with over 723,000 reads total and up to 140,637 reads recovered in just one sample (Supplementary Table S1). Because of these observations and the CPRJ’s proximity to a previous plague focus, we could not rule out a *Y. pestis* infection at this point. Official guidelines support that, in order to confirm a plague, it is necessary to have a positive culture or positive results in both serological and molecular tests. In this manner, no *Y. pestis* growth was observed, all samples showed no seroreactivity in the hemagglutination/ELISA tests performed by the IAM—Fiocruz, and no amplification was observed for *Y. pestis* specific genes *pla* and *caf1*. These results, associated with the higher prevalence of *Y. enterocolitica*, suggest that this pathogen was not the main source of this outbreak.

## 4. Discussion

In Brazil, zoonotic disease surveillance primarily targets mammals, and especially nonhuman primates (NHPs), which harbor a wide variety of pathogens [50]. In this study, we present an integrated epidemiological and genomic investigation of a toxoplasmosis outbreak that occurred in the CPRJ/Brazil with the first description of toxoplasmosis coinfection with bacterial sepsis.

Because of the security measures adopted due to the COVID-19 pandemic, the CPRJ had a significant reduction in the number of working employees, making all procedures take longer than usual. As the number of deaths grew rapidly, different tests were parallelized to deal with the situation. We first suspected of a SARS-CoV-2 infection, also considering the fact that two CPRJ staff members had reported going to work with respiratory symptoms; however, this theory was soon discarded. Rabies has a characteristic neurologic clinical picture that was not seen in any of the affected primates. However, YFV presents general clinical signs, such as prostration, loss of appetite, and dehydration [4], which were observed in the CPRJ NPs, but both pathogens were also discarded. Infections with other arboviruses, *Yersiniae*, hantavirus, and arenavirus were also investigated, considering the intestinal hemorrhage detected and CPRJ’s proximity to urban centers and forests, where there is a large circulation of wild rodents, but they were all dismissed.

In contrast, *T. gondii* DNA was detected in whole blood samples from all specimens evaluated. This biological sample was also used for the detection of parasitic DNA in a case of fatal toxoplasmosis in a free-living southern muriqui in São Paulo/Brazil [49], showing that it can be a strong indicator of acute phase of the parasitosis in recently infected hosts [51]. These results highlight the potential use of whole-blood PCR as a diagnostic tool, allowing for the immediate start of specific therapeutic managements, which may increase the survival of individuals. Although all animals died, the black-and-gold howler monkey (*A. caraya*, #8, ID 2576) began treatment just two days after the onset of clinical signs [48]. More details about the treatment can be found in the previous work, which is, to date, the first report of a NP that did not die acutely due to toxoplasmosis [48]. This was the only animal who showed IgG antibodies progression, and it was later euthanized, as there was no recovery of the general health status. It is possible that factors such as the difference in parasite load to which the individual was exposed in the captivity context added to the rapid administration of specific treatment for toxoplasmosis, contributing to the survival of this specimen. Another study also failed to detect anti-*T. gondii* antibodies in a captive *Callicebus nigrifons* (Pitheciidae) that died suddenly of toxoplasmosis in Brazil [52]. This absence of a humoral response becomes an important obstacle for its serodiagnosis.

Toxoplasmosis is a cosmopolitan infectious disease caused by protozoan *Toxoplasma gondii*, and it is one of the most common parasitic infections, although its prevalence varies widely depending on the location. It affects a wide variety of mammals and birds [6], causing either an acute or chronic clinical manifestation, but has felines as definite hosts, as they can shed resistant oocysts through their feces. Primates from CPRJ are kept in familiar groups and housed in outdoor enclosures built with mesh, with a natural floor covered with leaves, where they eventually encounter other sylvatic animals, besides mosquitoes and other insects. However, there were no reports of felines circulating in the center or in its surroundings, so an infection with *T. gondii* was not immediately suspected.

Primate diets are mostly composed of rations of fruits and vegetables, with an additional supply of local foliage for some species. As all affected animals were folivores, it is believed that infection may have occurred through contaminated foliage, which is purchased at local markets, where there is a large circulation of feral and stray cats, and they may not have been properly sanitized before consumption. This could explain why the affected animals were distributed in different enclosures. However, by the time when toxoplasmosis was detected, there was no foliage left to be analyzed. It is important to note that, as previously discussed [53], the strain of *T. gondii* isolated from the tissues of specimen 2576 (*A. caraya*, #8), for which inoculum in mice proved to be highly virulent [53], is genetically similar to the ones found in seven asymptomatic birds in Espírito Santo (ES) [54,55,56] and four human congenital toxoplasmosis cases in Minas Gerais (MG) in 2012 [56,57]. Both states border Rio de Janeiro, where the CPRJ is located.

Following national guidelines, testing majorly for YFV and rabies is one of the many bottlenecks for epizootic diagnosis. Strictly speaking, only diseases that appear on the National List of Compulsory Notification should be reported. This excludes a great number of other conditions, and even for notifiable ones, data are not widely and clearly available. Ideally, as we have a large number of agents that can affect animal populations, all notified cases should go through a wide investigation for multiple pathogens, but such an investigation is typically not performed. It is very rare for the community to perform those screenings, both because of the lack of appropriate funding for such high-cost experiments and the lack of trained staff or lab expertise. Importantly, considering that more than 24% of the NPs are considered endangered or worse according to the Red List of Threatened Species by the IUCN [58], their conservation should be a key concern.

Therefore, diseases that threaten animal survival and those that pose risks to humans should both be closely monitored. The low interest of public authorities in animal diseases other than YF and rabies results in long delays in redirecting negative samples to other laboratories, thus compromising diagnosis. The joint action between reference laboratories and research institutions, especially in those cases that do not fit into the hall of traditionally investigated diseases, allows for a faster response, which could increase the chances of survival. As soon as cases began to occur, and especially once the disease was identified, the measures taken were essential to prevent other animals from also becoming infected, which included mainly the cleaning of nurseries, an increase in biosecurity measures, and the replacement of the entire diet with fresh, recently sanitized food. These actions must be incorporated as preventive measures to avoid new cases. Thus, a collective and interdisciplinary response was essential to deal with this outbreak, and the rapid identification of the causative agent allowed for the implementation of innovative treatments that contributed to ameliorate the clinical outcome for the last affected animal.

## 5. Conclusions

This investigation concluded that the outbreak was caused by *T. gondii* 48 h after necropsy procedures and is the first description of toxoplasmosis coinfection with bacterial sepsis. We emphasize that gross examination and histopathology should be the starting choice for a diagnosis flowchart, while IHQ and PCR should be employed for the quick etiological confirmation. Diagnosis for toxoplasmosis is not included in the hall of zoonotic diseases investigated under official guidelines. Our study showcases a cross-platform interdisciplinary investigation to detect pathogens with public health relevance that are not included in current diagnostic policies, suggesting that the ongoing model of testing mainly for YFV and rabies presents important flaws and could be improved. Current public health policies on nonhuman animal disease notifications could make full use of existing interdisciplinary outbreak investigation approaches within a One Health framework.

## Figures and Tables

**Table 1:** Main pathological findings in neotropical nonhuman primates.

NHP		Species	Sex	Age *	Body Condition Score **	Hepatomegaly	Splenomegaly	Lymphadenomegaly	Hemorrhages					Necrosis	
									Lungs	Liver	Lymph Node	Small Intestine	Large Intestine	Stomach	Intestine
Atelidae	1	*B. arachnoides*	M	6	-	+	+	+	+	+	+	+	+	-	+
	2	*B. arachnoides*	F	20	4	+	+	+	+	+	+	+	-	-	+
	3	*A. ululata*	F	Adult	-	+	+	+	+	-	+	+	+	-	-
	4	*A. ululata*	F	Adult	-	+	+	+	-	-	+	-	-	-	-
	5	*A. ululata*	F	Adult	3	+	+	+	+	+	+	-	-	-	-
	6	*A. guariba*	M	11	3	-	+	-	-	-	-	-	-	-	-
	7	*A. guariba*	M	7	3	-	-	-	-	-	-	-	-	-	-
	8	*A. caraya*	M	10	2	-	+	+	-	+	+	-	-	-	-
Pitheciidae	9	*C. melanocephalus*	F	12	2	-	+	+	-	-	+	-	-	-	-
	10	*C. melanocephalus*	M	Adult	-				+	-	-	-	-	-	-
	11	*P. caligatus*	F	4	3	+	+	+	+	+	+	+	-	+	+

(M) male; (F) female; * years; (-) absent; (+) present; ** 1-to-5 scale.

**Table 2:** Histological findings in neotropical nonhuman primates with toxoplasmosis.

	Atelidae. Affected Monkeys/Severity				Pitheciidae. Affected Monkeys/Severity	
	*B. arachnoides* (n = 2)	*A. ululata* (n = 3)	*A. guariba* (n = 2)	*A. caraya* (n = 1)	*C. melanocephalus* (n = 2)	*P. caligatus* (n = 1)
***Liver***						
Lymphocytic periportal hepatitis	2/++	3/++	2/+	1/++	2/++	1/+++
Random necrosis	2/++	3/++	2/+	1/++	2/++	1/++
Hemorrhage	1/+	2/+			1/+	1/+
Fibrin deposition	2/++				1/+	-
Hemosiderosis	1/+	1/+		1/+	1/+	
Steatosis		2/+++	2/+++		1/++	1/+++
***Lung***						
Interstitial pneumonia	2/++	2/++	2/+	1/++	2/++	
Edema	2/++	2/++	2/+++	1/++	2/+++	1/+
Hemorrhage	2/++	2/++	1/+		1/+++	1/+++
Fibrin deposition	1/+	2/++	1/+		1/++	1/+
Lymphocytic perivascular infiltrate		1/++			1/+++	1/+++
***Lymph Node***						
Necrotic lymphadenitis	1/+++	2/+++			2/++	1/+++
Hemorrhage	1/++	2/+++		1/++	1/++	1/+++
Hemosiderosis	1/+++				2/++	1/+++
***Spleen***						
Necrotic splenitis	1/++	2/++	2/++			
Hemorrhage	1/+++	2/+	1/+		1/++	1/+++
Fibrin deposition	1/+++	2/+	1/++		1/+	1/+++
***Alimentary system***						
Necrohemorrhagic gastritis						1/+++
Necrohemorrhagic duodenitis	2/+++					1/+++
Necrohemorrhagic jejunitis						1/+++
Necrohemorrhagic typhlitis	1/+++					
***Brain***						
Multifocal malacia	-	2/++			1/+	
Hemorrhage	-					
White matter	-	2/+			1/+	1/+++
Gray matter	-	2/+			1/+	1/+++

(+) mild; (++) moderate; (+++) severe. (-) absent.

**Table 3:** Immunohistological findings in neotropical nonhuman primates with toxoplasmosis.

NHP		Species	Sex	Age *	Anti-*T. gondii* (Liver and Lung)		Anti-*SARS-CoV* (Lung)	Anti-*E. coli* (Lung)				
					Bradyzoite Cists	Tachyzoites		Type I Pneumocyte	Type II Pneumocyte	Alveolar Septum	Endothelium	Vascular Lumen
Atelidae	1	*B. arachnoides*	M	6	+	+	−	+++	−	++	+++	+
	2	*B. arachnoides*	F	20	+	+	−	+++	−	−	++	++
	3	*A. ululata*	F	Adult	+	+	−	+	−	−	−	−
	4	*A. ululata*	F	Adult	+	+	−	+++	−	−	++	++
	5	*A. ululata*	F	Adult	+	+	−	+++	−	−	+++	+++
	6	*A. guariba*	M	11	+	+	−	NP	NP	NP	NP	NP
	7	*A. guariba*	M	7	+	+	−	NP	NP	NP	NP	NP
	8	*A. caraya*	M	10	−	−	−	NP	NP	NP	NP	NP
Pitheciidae	9	*C. melanocephalus*	F	12	+	+	−	+++	−	−	++	−
	10	*C. melanocephalus*	M	Adult	+	+	−	NP	NP	NP	NP	NP
	11	*P. caligatus*	F	4	+	+	−	+++	−	−	++	++

(M) male; (F) female; * years. For “Anti-*T. gondii*”: (−) negative; (+) positive. For “Anti-*E. coli*”: (+) mild; (++) moderate; (+++) severe.

## Data Availability

The raw sequencing data generated in the study were submitted to the Sequence Read Archive (SRA) databank from the NCBI server, under the BioProject number PRJNA986486 and BioSamples numbers SAMN35839772 to SAMN35839791. Other relevant data are contained within the manuscript or available at https://github.com/lddv-ufrj/CPRJ_Outbreak (last accessed on 19 June 2023).

## Supplementary Materials

The following supporting information can be downloaded at https://www.mdpi.com/article/10.3390/microorganisms11122888/s1; Figure S1. Taxonomic diversity in NP samples classified by Kraken; Table S1. Main findings from the reference assembly, using metagenomic data.
