# Supplementary material for: Lessons from a Multilaboratorial Task Force for Diagnosis of a Fatal Toxoplasmosis Outbreak in Captive Primates in Brazil
Source: Microorganisms. 2023 Nov 29;11(12):2888. doi: 10.3390/microorganisms11122888 (PMC10745312; doi:10.3390/microorganisms11122888)
Supplement: Supplementary file 1 [file microorganisms-11-02888-s001.zip › microorganisms-2701223-supplementary.pdf]

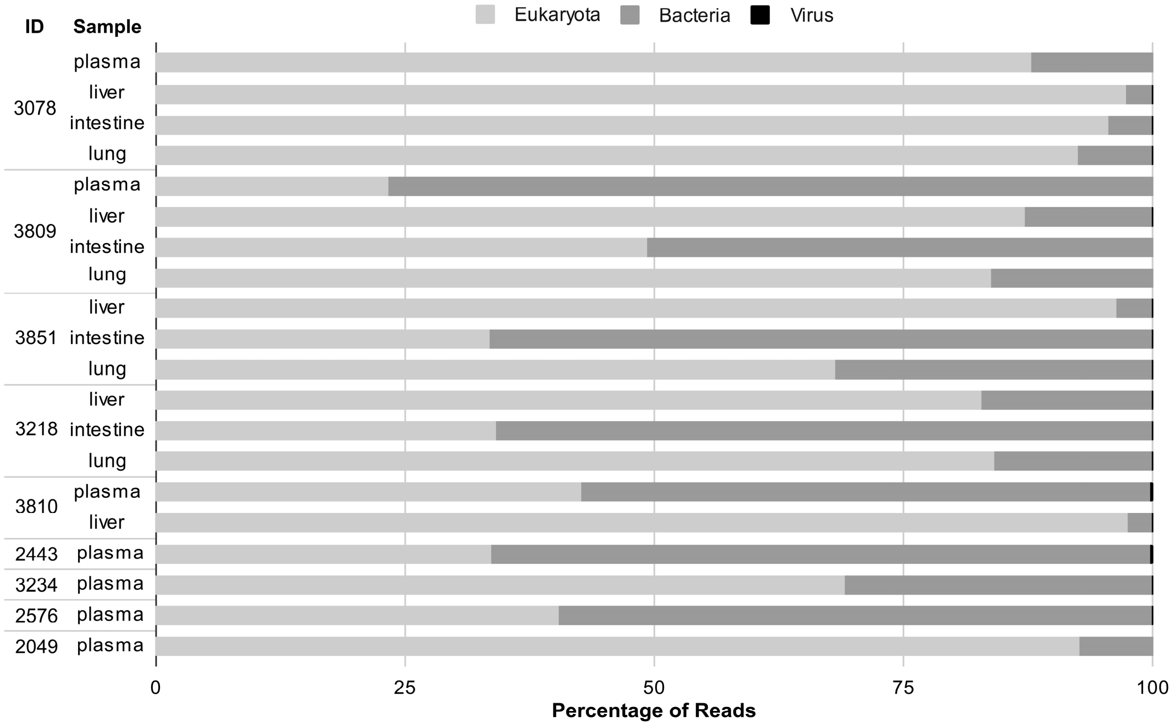

Figure S1. Taxonomic diversity in NP samples classified by Kraken. Sequences from NP samples collected during the outbreak were classified using Kraken with the miniKraken_v2 database. Eukaryotic reads are colored in light gray, bacterial reads in dark grey and viral reads in black.

Table S1. Main findings from the reference assembly using metagenomic data.

NHP	Sample	*SARS-CoV-2* NC_045512.2		*Yersinia pestis* AE017042.1		*Yersinia enterocolitica* NC_008800.1		*Yersinia pseudotuberculosis* NZ_LR134373.1		*Plasmodium brasilianum* GCA_001885115.2		*Toxoplasma gondii* NC_031467.1		*Salmonella enterica* AE006468.2	
3078	Plasma	0	0%	6.111	3%	19.694	3%	17.001	3%	10.460	3%	7.084	3%	6.156	3%
	Kidney	0	0%	1.797	1%	5.396	1%	5.365	1%	73.759	1%	**109.870**	**1%**	1.875	1%
	Intestine	0	0%	1.942	1%	5.681	1%	5.935	1%	32.116	1%	41.541	1%	2.051	1%
	Lung	0	0%	4.806	2%	14.648	2%	14.084	2%	60.852	2%	50.119	2%	4.830	2%
3809	Plasma	0	0%	26.068	11%	74.526	11%	82.353	13%	32.975	13%	10.308	13%	26.462	13%
	Kidney	1	4%	28.046	12%	86.813	12%	82.069	13%	37.246	13%	47.782	13%	28.185	13%
	Intestine	0	0%	4.041	2%	11.378	2%	12.757	2%	14.124	2%	28.818	2%	4.038	2%
	Lung	0	0%	7.917	3%	24.604	3%	22.914	4%	16.375	4%	10.302	4%	8.086	4%
3851	Kidney	0	0%	1.998	1%	5.966	1%	5.996	1%	81.988	1%	67.331	1%	2.029	1%
	Intestine	0	0%	19.284	8%	57.009	8%	58.115	9%	15.260	9%	12.359	9%	18.990	9%
	Lung	0	0%	3.051	1%	9.742	1%	8.643	1%	89.338	1%	24.006	1%	2.969	1%
3218	Kidney	0	0%	12.015	5%	38.253	5%	34.947	5%	45.015	5%	20.672	5%	12.179	5%
	Intestine	1	4%	**40.532**	**18%**	**140.637**	**18%**	**105.136**	**16%**	11.827	16%	16.674	16%	**41.752**	**16%**
	Lung	0	0%	10.171	4%	30.576	4%	30.523	5%	72.098	5%	46.862	5%	11.091	5%
3810	Plasma	0	0%	7.342	3%	24.581	3%	19.498	3%	13.953	3%	8.907	3%	7.135	3%
	Kidney	2	8%	1.678	1%	5.428	1%	4.634	1%	**99.572**	**1%**	107.929	1%	1.714	1%
2443	Plasma	0	0%	25.921	11%	78.574	11%	77.393	12%	27.271	12%	13.883	12%	26.398	12%
3234	Plasma	0	0%	1.296	1%	4.137	1%	3.649	1%	2.580	1%	1.659	1%	1.295	1%
2576	Plasma	**21**	**81%**	15.169	7%	55.802	7%	35.995	5%	30.484	5%	6.337	5%	15.794	5%
2049	Plasma	1	4%	9.502	4%	29.859	4%	27.448	4%	50.209	4%	28.251	4%	9.620	4%
